# Can emotions be professional? A decolonial reframing of professional identity formation

**DOI:** 10.3389/fmed.2026.1952561

**Published:** 2026-10-16

**Authors:** Sofia Camargo Collet, Ligia Maria Cayres Ribeiro, Wieke E. van der Goot, Marco Antonio de Carvalho Filho, Francisco Manuel Olmos-Vega

**Affiliations:** DVOO - Cross-Connection Education and Training, LEARN – Lifelong Education and Assessment Research Network, University Medical Centre Groningen, University of Groningen, Netherlands

**Keywords:** decolonialism, emotions, professional identity formation, professionalism, transformative learning

## Abstract

Medical professionalism has long been associated with rationality, emotional detachment, and self-control. Although often presented as universal standards of professional conduct, these norms are historically shaped by Western and individualistic epistemologies that frame emotions as threats to objectivity and ethical judgment. Such expectations shape Professional Identity Formation (PIF), often generating tensions between learners’ lived experiences and institutional norms. We argue that emotions are essential resources that qualify decision-making by helping us understand ourselves, the other, and the context, and anticipate the consequences of our decisions, thereby being essential for learning and professional identity formation. We draw on three decolonial perspectives. First, research on queer identities shows how trainees act as care weavers, transforming their own experience of marginalization into a relational capacity for empathy and clinical insight. Second, the South African philosophy of Ubuntu positions compassion and humanness not as soft skills but as constitutive markers of professional conduct grounded in communal interdependence. Third, rooted in Freire's critical pedagogy, the Pedagogy of Connection defines professionalism through presence—an openness to patients’ suffering that qualifies clinical reasoning and positions the physician as an agent of social justice. Drawing on transformative learning theory, we suggest that emotionally challenging experiences can prompt reflection and shifts in perspective that are central to professional identity formation. Building on these perspectives, we propose reconnecting professionalism with emotions. We argue that emotional engagement is constitutive of professional identity formation, while its expressions and legitimate forms are shaped by the contexts in which physicians learn and practice.

## 1 Introduction

Medical professionalism is conceptualized as a collective promise, a social contract between the medical profession and society, encompassing the values, behaviors, and relationships that sustain public trust (1, 2). Yet it has long been associated with rationality, emotional detachment, and self-control. These associations have roots in a Western colonial tradition (3) and in a Cartesian separation of emotion from reason, which portrays feelings as a corruption of objective clinical knowledge (4). The consequence of this division is that trainees are socialized to act with detached concern (3), systematically suppressing their emotional reactions to remain objective (5, 6).

In clinical training, this framing becomes particularly visible when trainees encounter emotionally complex situations. Trainees are exposed to death, uncertainty, suffering, and systemic injustice at a stage when they are still developing their professional identities. These experiences often evoke vulnerability, explained as stepping beyond one's comfort zone into situations of uncertainty, risk, and limited control (7). For many trainees, vulnerability is also shaped by the educational and social contexts in which they learn. Experiences of marginalization, exclusion, discrimination, and mental burden can intensify this vulnerability, particularly for learners from underrepresented backgrounds. For example, Ribeiro et al. (6) describe how trainees in end-of-life care are shocked by the physical realities of death while navigating a culture that treats dying as taboo and values emotional distance as the appropriate professional response. Likewise, van Duin et al. (7) show that entering clinical practice places learners in unfamiliar situations where uncertainty, responsibility, and fear of being judged incompetent make vulnerability inevitable. In the Brazilian context, daily encounters with structural inequities similarly leave trainees feeling powerless as they witness the suffering of marginalized populations (8). Despite these challenges, trainees are implicitly expected to manage these experiences alone, through individual emotional restraint (3, 9).

When these emotionally charged experiences cannot be openly acknowledged, they may give rise to shame. Shame transforms emotional distress into negative judgments about the self, leading trainees to interpret vulnerability as evidence that they are inadequate or do not belong in medicine rather than as a natural response to complex clinical practice (10). Medical education can become an environment fruitful for shame, where hierarchical relationships, psychologically unsafe learning environments, perfectionism, and experiences of underrepresentation increase the likelihood and intensity of shame experiences (11, 12)^.^ In this context, many trainees adopt a cloak of competence, hiding their emotions and struggles to avoid being perceived as unprofessional or illegitimate (13). Instead of supporting reflection and growth, shame reinforces emotional suppression, identity dissonance, and isolation, shaping how trainees come to understand themselves as future physicians (10, 13)^.^

Rivas et al. found that trainees frequently hold the implicit assumption that being a “good doctor” requires suppressing emotions, a process that progressively reduces their awareness of their own feelings and those of others (14). To counteract the consequences of a lack of emotional awareness, innovative educational initiatives have successfully used music to promote emotional education in medical training (14, 15). By creating space for emotional reflection and expression, these interventions help normalize emotions within medical practice, strengthen trainees’ emotional awareness, reconnect them with the values that motivated them to pursue medicine, and prepare them to build more meaningful relationships with patients in their future practice.

Although emotional education has demonstrated important benefits for learners, it remains the exception rather than the norm. Medical Professional Identity Formation (PIF) continues to be shaped by dominant expectations of emotional detachment, emotional suppression, and the solitary management of emotions. PIF is the ongoing process through which medical trainees come to think, act, and feel like physicians (10, 11). Medical professionalism shapes this process by defining the external framework of values and behaviors trainees are expected to embody (1, 9). When professionalism privileges emotional distance, it reflects perspectives largely rooted in Western societies of the Global North, where cultural norms shape how emotions are experienced, expressed, and discussed in everyday life. These assumptions become embedded in medical education and influence what is considered appropriate professional behavior (16). As a result, trainees may struggle to reconcile their own emotions and values with expectations of emotional detachment, creating identity dissonance (6)˒—the distress of being unable to align one's personal values with a detached professional role (6, 12). To navigate this tension, many conceal vulnerability and uncertainty to avoid appearing unprofessional or inadequate (3, 9). Over time, these unresolved tensions can lead to isolation, burnout, cynicism, and a diminished sense of purpose (6–8).

These tensions invite us to question the assumptions of dominant understandings of medical professionalism. In this article, we adopt a decolonial perspective to critically examine these assumptions, recognizing that professionalism is not a neutral or universal concept but is shaped by historical, cultural, and political contexts (2, 4). Rather than treating existing norms as fixed standards to which all trainees should conform (11, 12), a decolonial perspective encourages us to ask whose values, ways of knowing, and ways of relating have been privileged, and whose have been marginalized or excluded (2, 13). From this standpoint, professionalism becomes a culturally situated and negotiated social contract that can be reimagined through multiple ways of knowing, relating, and becoming a physician (16–18).

Therefore, in this perspective article, we argue that emotions should not be treated as disruptions to professionalism but as essential resources for learning and PIF (3). We reject the Cartesian view that positions emotion and reason as opposing forces, recognizing instead that they are deeply intertwined in shaping clinical judgment, reflection, and professional growth (5, 8).

Importantly, Western thought is not monolithically Cartesian; existential psychology, grounded in the phenomenological tradition (e.g., Jaspers, Heidegger, Frankl), offers a vital counter-perspective by framing emotions, anxiety, and vulnerability in ‘limit situations’ (such as confronting death and severe uncertainty) as fundamental, embodied ways of knowing rather than irrational noise. However, because classical existential models originate in European contexts, they often carry universalist and individualistic assumptions that risk abstracting emotion from local, social and historical structures. Therefore, we acknowledge this Western tradition while employing a decolonial lens to ground these emotional ways of knowing within specific cultural, relational, and structural realities (19).

In the following sections, we draw on queer perspectives, Ubuntu philosophy, and critical pedagogy to broaden current understandings of professionalism. We then turn to transformative learning theory to explain how vulnerability, emotional tensions, and disorienting dilemmas can become powerful catalysts for learning and professional identity formation.

## 2 Discussion

### 2.1 Decolonial perspectives on professionalism

To reconnect professionalism with emotions, we explore three complementary perspectives that broaden dominant understandings of professionalism. First, Queer perspectives on PIF are rooted in different philosophical and theoretical fields, including Queer Theory, Intersectionality Theory, and broader Critical Theory perspectives. Given the diversity of theoretical underpinnings, queer perspectives challenge the dominant norms of gender and sexuality, and illustrate how these norms create systems of oppression and privilege, inclusion and marginalisation of groups in social and professional societies. Beyond critiquing these norms, queer scholarship advances a distinct epistemological claim: that emotion is itself a way of knowing rather than an obstacle to knowledge (20). Ahmed argues that the hierarchy privileging reason over feeling is not epistemically neutral, but historically bound to colonial logics that associated emotion with the primitive and the racialised other (20). Read this way, the emotional detachment demanded by dominant professionalism is not only a Cartesian inheritance but a colonial one, and the queer critique of that hierarchy is continuous with the decolonial argument we develop here.

Research focusing on queer identities in medical education illustrates how individuals act as care weavers, drawing on lived experiences of marginalization to transform personal vulnerability into a relational capacity for empathy and clinical intelligence (21). For these trainees, marginalization acts as a lifelong disorienting dilemma that prompts a transformative rejection of dehumanizing professional norms in favor of radical connection (21). By experiencing exclusion, precarity, and mental burden in their own lives, their own vulnerability becomes a means of connecting with patients, who are themselves rendered vulnerable by illness; they redefine professionalism as an act of resistance that centers relational safety and social consciousness as core ethical duties (18, 21). What queer trainees develop here is not only a disposition but an epistemic standpoint. Queer epistemologies hold that knowledge is produced through embodied and affective experience, and that the feelings generated by living athwart a norm — discomfort, disorientation, shame — disclose how that norm operates in ways that are not available from within it ^(^20, 22)^.^ Sedgwick's account of shame is instructive here: shame is not only a wound to be healed but a relational, world-disclosing affect through which the self becomes legible to itself and to others (23). This reframing matters for our argument, because it positions the emotional knowledge queer trainees bring to clinical encounters as a form of expertise rather than as compensation for exclusion. We are careful, however, not to treat marginalisation as productive in a way that would justify its harms. Our claim is not that exclusion is educative, but that knowledge generated under conditions of exclusion should be recognised as knowledge rather than discounted as personal experience.

This capacity developed by queer trainees resonates with bell hooks’ understanding of the margin (24) as more than a place of exclusion or deprivation. Living at the margins creates a particular way of seeing because it requires understanding both the dominant structures of society and the realities of those positioned outside them. This perspective makes visible assumptions and forms of oppression that often remain unnoticed from the center, while opening possibilities for imagining alternatives (24). Rather than viewing the margin only as a place of suffering, hooks argues that it is also a space of resistance, creativity, and transformation, where new ways of knowing and being can emerge (24). In other words, by accessing their vulnerabilities, queer trainees open space to connect with the other; even when the other is very different from them, they recognize vulnerability as a shared human condition, which is an essential skill to offer quality clinical care, built from a place of marginalization (21).

Recognizing vulnerability as a shared human condition is also a central premise of the South African philosophy of Ubuntu (18). More than an ethical framework, Ubuntu represents a relational ontology that understands personhood through the principle “*I am because we are"* (18) Human beings become who they are through their relationships with others, making identity an ongoing and collective process of becoming. This understanding extends to professional identity, positioning the process of becoming a physician as inseparable from relationships with patients, colleagues, communities, and society.

This relational understanding also shapes the role of emotions within professionalism. In Ubuntu, emotions are not understood primarily as private inner states belonging to an isolated individual. They are better understood as relational forces: they disclose, sustain, and repair the bonds through which people connect, care for one another, and learn from one another. Emotion is therefore not a private matter to be managed but a shared matter to be attended to — which is precisely what the dominant model, with its individualized ethic of self-control, cannot accommodate. Compassion, empathy, kindness, and emotional presence allow physicians to recognize their shared humanity with patients and colleagues. One can compare Ubuntu to a hand, in which each finger has its own role while remaining dependent on the others to function. Likewise, physicians develop their professional identities through relationships, where listening to patients’ stories, acknowledging vulnerability, and remaining emotionally present become expressions of professionalism (25). Emotions therefore sustain these relationships, creating opportunities for connection, reflection, and professional growth. Emotion as a way of knowing implicates that epistemologically, clinical truth and personhood are not objective facts extracted by an isolated observer, but are relational understandings co-constructed through communal interdependence. Because African epistemology starts with the community and moves to the individual, emotions do not function as private inner psychological states to be managed in isolation (17, 18, 25). Instead, they act as relational forces that disclose, sustain, and repair human connection. Epistemologically, knowing a patient requires going beyond disease pathology to listen to their story within their communal context. In this framework, emotional presence and mutual vulnerability become necessary perceptual tools to know and respect the person behind the medical diagnosis.

By sustaining relationships, emotions also create the conditions for solidarity. Within Ubuntu, solidarity reflects the recognition that our well-being and responsibilities are fundamentally interconnected, creating a shared commitment to care for one another and for the communities we serve (18). Professionalism therefore becomes a collective practice grounded in compassion, mutual responsibility, and shared humanity. This relational understanding provides a foundation for emotional engagement to support transformative learning and professional identity formation.

If Ubuntu locates professionalism in relationship, critical pedagogy locates it in structure. The relational commitments Ubuntu describes cannot be willed into being by individual practitioners; they require educational and health systems that make them possible — and they are actively obstructed by systems that do not. Professionalism as a relational process therefore also depends on the educational structures in which it develops. Educational environments shape how learners experience relationships, engage with emotions, and understand their responsibilities toward patients, communities, and society. According to Freire's critical pedagogy (26), in which dialogue and the reading of one's social world are the conditions for critical consciousness, learners are encouraged to connect their emotional experiences to the historical, social, and institutional contexts that shape healthcare, thereby developing a sense of agency and responsibility in contributing to change (26, 27). Professionalism therefore grows through educational experiences that intentionally connect learners with the healthcare system, the communities they serve, and the values of social justice. Inspired by Critical Pedagogy and drawing on the Brazilian context, de Carvalho Filho and Hafferty propose a Pedagogy of Connection that emerged from the close integration between medical education and the country's public healthcare system (8). An example of such integration comes from Brazil, where, from the beginning of their training, students participate in community and primary care settings, encountering the complexity of patients’ lives, the realities of social inequality, and the challenges of delivering equitable healthcare. These experiences provide the context in which professionalism is learned, and professional identity begins to develop. These early patient encounters expose learners to situations that can evoke uncertainty, frustration, and a sense of powerlessness as they recognize that many health problems are rooted in broader social and structural conditions. The Pedagogy of Connection responds by creating educational spaces where these experiences can be explored collectively through dialogue and critical reflection (8).

In this Pedagogy of Connection, emotion as a way of knowing implicates that epistemologically, clinical practice cannot be divorced from emotional presence. Presence involves an emotional, rational, and spiritual openness to patients’ suffering, allowing emotions to inform decision-making and attention to focus on the human consequences of care. This openness becomes particularly important when trainees encounter structural inequities (8). Feelings of frustration, powerlessness, or moral outrage can reveal conditions that extend beyond the individual clinical encounter, making systemic problems visible. Martín-Baró's (28) liberation psychology helps explain this connection between emotional experience and structural conditions. Drawing on Freire's concept of critical consciousness, Martín-Baró understands emotional distress in limit situations not only as an individual psychological experience, but also as a response to the social conditions in which people live (28). From this perspective, emotions can help trainees recognize how their experiences are connected to broader structures and injustices, contributing to the development of critical consciousness (28). Gupta further shows how expressing and sharing these experiences, including through creative and arts-based practices, can challenge institutional cultures of silence (29). Emotions then become part of a collective process of making sense of oppression and moving toward collective action (28, 29). Educational structures that promote connection, dialogue, and critical reflection create opportunities for learners to make sense of emotionally significant experiences together. Crucially, this making sense process relies on treating emotion as an epistemological stance, a legitimate way of knowing: when emotions are recognized as perceptual tools that yield diagnostic and structural insights, affective discomfort becomes the primary guide for critical reflection. Through this process, assumptions about themselves, their patients, and the profession are gradually re-examined, allowing professional identity to evolve over time. This process of reinterpreting experience through reflection and dialogue forms the foundation of transformative learning.

Together, these perspectives reframe professionalism as a process that develops across emotional, relational, and structural dimensions of medical education. Vulnerability becomes a starting point for learning by inviting learners to engage with uncertainty and their shared humanity. Relationships provide the context in which these experiences acquire meaning, connecting learners with patients, colleagues, communities, and themselves. Educational structures shape how these relationships are experienced by creating spaces for dialogue, reflection, and critical engagement with the healthcare systems and societies in which medicine is practiced. Professional identity, therefore, develops through the continuous negotiation among emotions, relationships, and the broader contexts in which learning takes place.

This broader understanding of professionalism also expands the sources from which medical education can draw knowledge. The perspectives discussed throughout this paper illustrate the possibility of drawing from the margins where new ways of knowing can emerge. By bringing vulnerability, relationality, and structural responsibility into discussions of professionalism, they reveal dimensions of becoming a physician that have remained less visible within dominant models of medical education. Learning from the margins, therefore, expands not only whose voices are heard but also how professionalism itself is understood, taught, and lived.

### 2.2 Transforming emotional tension into learning

The perspectives discussed above suggest that experiences of vulnerability, relationships with others, and engagement with educational and social structures are central to becoming a physician. Transformative learning theory provides a framework for understanding how these emotionally significant experiences can challenge existing assumptions and become opportunities for learning and professional identity formation. Transformative learning begins with a disorienting dilemma, an experience that challenges previously held assumptions and frames of reference, prompting critical reflection (30, 31). This process of critical reflection requires learners to become aware of their own systems of beliefs and values, question how these systems shape their interpretations, and how they could remain open to alternative perspectives (32). Importantly, transformative learning engages both cognitive and affective dimensions: it involves not only rational reflection but also the capacity to recognize, examine, and learn from one's emotions, attitudes, and lived experiences (30). Through this integration, learners can develop new ways of understanding themselves, others, and the world (30, 31).

In health professions education, clinical settings provide a unique context for transformative learning, as trainees routinely encounter emotionally and ethically complex situations that may challenge their existing assumptions (6, 7, 10, 32). As discussed earlier, experiences such as first encounters with end-of-life care or reaching the limits of one's own capabilities can leave trainees feeling overwhelmed, powerless, uncertain, and alone, unsettling their assumptions about the process of dying, care, and their professional role (6, 7). Rather than representing signs of incompetence, these emotional responses can become disorienting dilemmas that prompt critical reflection and shifts in perspective when learners are supported in acknowledging, making sense of, and expressing their emotions (7).

By reframing medical professionalism as a holistic, transformative process of clinical learning, we can move away from the dominant Cartesian view of professionalism as emotional detachment and self-control, in which trainees are encouraged to suppress rather than engage with these experiences (3, 9).

## 3 Conclusion

We propose a new conceptual approach to medical professionalism that reconnects professionalism with emotions by recognizing vulnerability as a source of learning, relationships as central to professional identity, educational spaces as sites for dialogue and critical reflection, and transformative learning as a process of collective meaning-making. We do not offer this as a new universal standard to replace the old one. We argue instead that emotional engagement is constitutive of professional identity formation, while the meanings, expressions, and legitimate forms of that engagement are shaped by the cultural, structural, and historical contexts in which physicians learn and practice. Emotions connect learners to themselves through reflection, to patients and colleagues through relationships of care, and to the broader social realities of healthcare through solidarity and social responsibility. In doing so, professionalism becomes a relational process of becoming that extends beyond the individual, recognizing that physicians are shaped through their connections with others, the communities they serve, and the systems in which they practice. By positioning emotion as a legitimate way of knowing, feelings are recognized not as subjective distractions from clinical practice, but as perceptive tools that deepen understanding.

Relational professionalism calls for educational spaces where emotionally challenging experiences are explored collectively rather than carried individually. Learning from the margins means creating spaces of radical openness where vulnerability, uncertainty, and emotional tensions are recognized as shared opportunities for reflection rather than personal shortcomings. Through dialogue, critical reflection, and collective meaning-making, these experiences can become catalysts for transformative learning, enabling learners to cultivate professional identities grounded in connection, solidarity, and social responsibility. In this way, professionalism becomes not only a framework for regulating conduct, but also a pedagogical practice that supports learners in becoming physicians together.

## Data Availability

The original contributions presented in the study are included in the article/Supplementary Material, further inquiries can be directed to the corresponding authors.
